# Tunable mode control through myriad-mode fibers

**DOI:** 10.1109/jlt.2021.3057615

**Published:** 2021-02-08

**Authors:** Sakshi Singh, Simon Labouesse, Rafael Piestun

**Affiliations:** Department of Electrical, Computer, and Energy Engineering, University of Colorado Boulder, Colorado 80309, USA.

**Keywords:** Multimode fibers, wavefront shaping, mode control

## Abstract

Multimode fibers are attractive for imaging, communication, computation, and energy delivery. Unfortunately, intermodal and polarization coupling precludes direct control of the delivered mode composition. We present a technique to tailor the mode composition at the output of a multimode fiber with thousands of modes, which we refer to as myriad-mode fiber, using its experimentally measured transmission matrix. While precise mode control has been demonstrated in typical multimode fibers with up to 210 modes, the method proposed here is particularly useful for high mode number fibers, such as when the number of modes is comparable to the number of modes of the wavefront shaping spatial light modulator. To illustrate the technique, we select different subsets of modes to create focal spots at the output of a fiber with 7140 modes. Importantly, we define efficiency and fidelity metrics to evaluate the mode control and demonstrate the relationship between efficiency, fidelity, and the spatial location of the spots across the distal fiber cross-section.

## I. Introduction

MULTIMODE fibers (MMFs) have found application in classical [1], [2], [3], [4] and quantum communication [5], high dimensional quantum key establishment [6], [7], [8], transport of spatially entangles qubits [9], conservation of orbital angular momentum [10], sensing [11], [12], [13], energy delivery [14], [15], [16], computation [17], [18], phase conjugation [19], [20], [21], [22]and imaging[23], [24], [25]. Particularly interesting is the recent demonstration of ultrathin imaging endoscopes via wavefront shaping control to compensate for the effects of dispersion and mode coupling [26], [27], [28].

All these applications are enabled by some form of control over the modes of the fiber. Recently, spatial light modulators (SLMs) have been used for controlling all the modes in the fiber mode basis of typical multimode fibers with up to 210 modes [29], [30], [31]. However the method requires precise alignment, polarization control, and a number of SLM pixels much greater than the number of modes in the fiber. On the other hand, large-core MMFs with thousands of modes, which we refer to as myriad-mode fibers (MyF) here, are better suited for imaging due to their efficient light collection and high-resolution imaging capability. Mode control in MyFs remains a major challenge due to the large number of degrees of freedom and the detrimental effects of inter-modal and polarization coupling [32].

Specifically, in the field of fiber-optic communication, MMFs hold significant interest due to their large information carrying capacity. The achievable data rates through MMF transmission systems however are still severely limited by modal dispersion, coupling and nonlinearities. Mode division multiplexing is a promising technique which allows using different modes of the MMF as separate information channels to enhance the fiber capacity. It has been demonstrated by offset launching [33], using phase plates or gratings [34], [35], photonic crystal fibers [36] or multicore fibers [37] by phase and amplitude modulation using spatial light modulators [38], [39] and multi-plane light conversion devices [31]. These techniques aim for precise control of individual fiber modes and are hence not easily scalable to MMFs supporting thousands of modes.

**Fig. 1.**
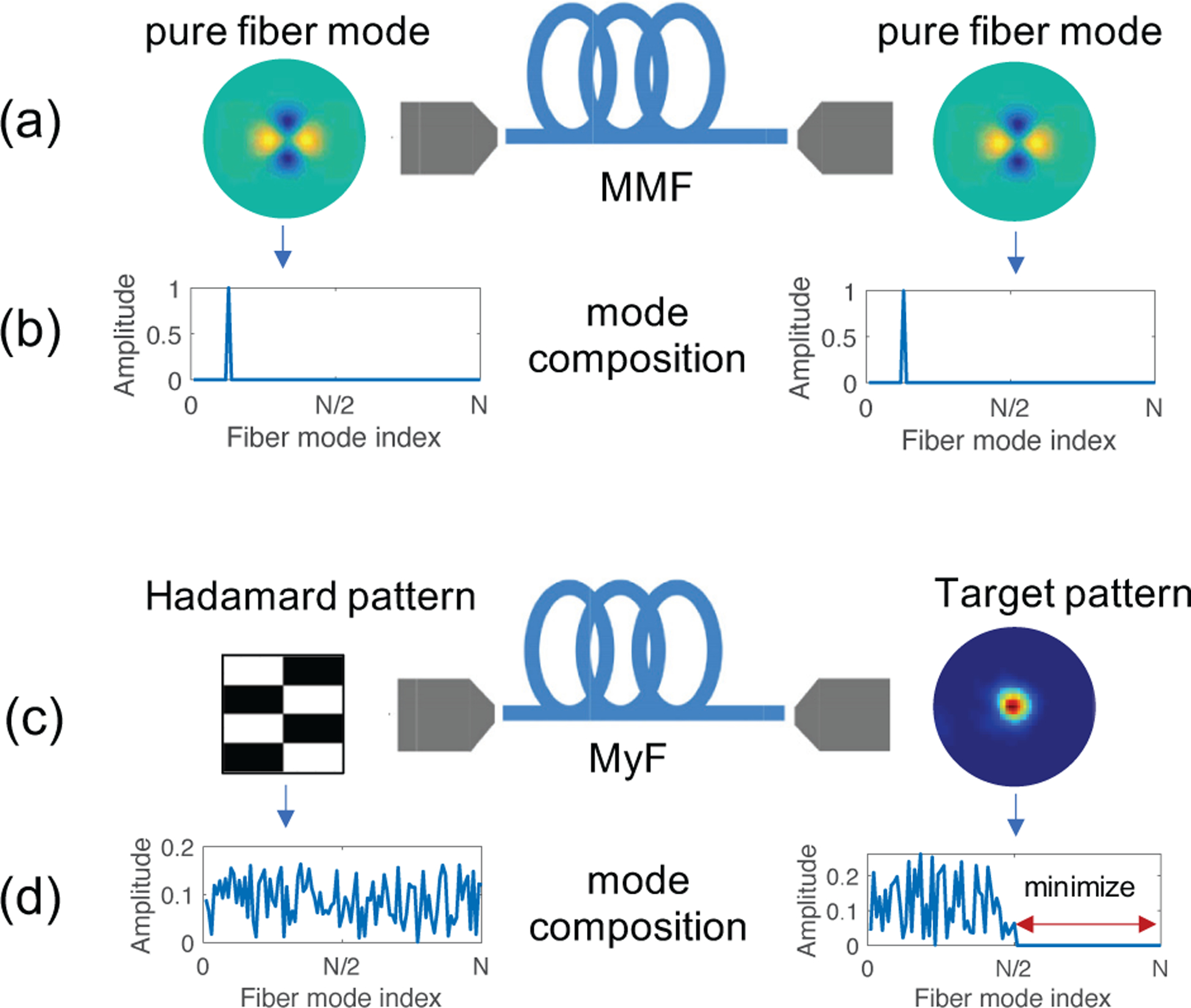
Illustration of methods for mode control in the fiber mode basis. (a) Mode control via excitation and generation of pure fiber modes. (b) Fiber mode composition of the input and output fields in (a). Since individual pure modes are excited and detected, their corresponding mode compositions are identical delta functions. (c) Proposed method for mode control in the fiber mode basis via excitation of Hadamard functions and detection of focal spots. (d) Fiber mode composition of the input and output fields in (c). Since Hadamard functions and focal spots are complex combinations of the individual fiber modes, their mode compositions are distinct complex signals. By performing a change of basis we can select the fiber modes that we control at the output and minimize the other mode coefficients. Here, as an example we minimize the second half higher order mode coefficients in order to generate a target output pattern using only the first half lower order modes.

In this report, we present a method to select specific groups of fiber modes at the output of an MyF using a phase-only spatial light modulator at its input. As an application example, the selected modes are constructively interfered at a predefined location in the fiber output, hence forming a focal spot. While we use the Hadamard basis at the input and generate focal spots at the output, we achieve mode control in the fiber mode basis via a digitally implemented basis transformation. Figure 1 illustrates the difference in methodology between the currently used techniques for fiber mode control through multimode fibers and our technique for fiber mode control through myriad mode fibers.

As a result, one can take advantage of the different properties of mode groups and their interactions. The fiber mode composition of the focal spot can be tuned by modifying the input pattern. Furthermore, we perform experiments to compare the sensitivity of focal spots to fiber bending when created using two different complementary mode sets. Towards a quantitative evaluation of the quality of mode control, we define specific efficiency and fidelity metrics that help understand the possibilities and limitations of mode control.

## II. Mode control in the fiber mode basis

Wavefront shaping is becoming a key technique in imaging and energy delivery through scattering media and MMFs. One of the preferred approaches involves characterization of the medium, in our case the fiber, through the transmission matrix (TM) [40], [29]. The measured TM, can be used to generate target field distributions at the fiber distal end such as focal spots. Here, we employ the phase-shifting interferometric approach for TM calibration using an internal reference frame [40], [41] and recover the output field using three intensity measurements. The calibration method is detailed in SI section 1.

Let us consider the problem of generating a physically feasible output field, *E*_*target*_, at the distal end of the fiber. *E*_*target*_ can be written as a superposition of all the fiber modes. Assuming a radially symmetric parabolic refractive index profile, the linearly polarized (LP) modes of a GRIN (graded-index) MyF can be represented using the Laguerre-Gauss (LG) field solutions [42], [43]. We note that the actual modes of the fiber will deviate from the LG mode profiles, depending on the true refractive index profile, imperfections and bend configuration of the fiber. Notwithstanding, to demonstrate the method here, we implement mode control in the LG mode basis as they are a very good approximation of the actual modes.

For an MyF with *N*_*m*_ number of modes per polarization, stored in the columns of a matrix, F, which we call the mode matrix, we can represent an *N*_*out*_- pixel *E*_*target*_ field in the modal basis by taking its product with the inverse of the F matrix. However, F can only be invertible when it is a square matrix, which is true only when the number of samples in *E*_*target*_ is equal to the number of fiber modes. In the experiments described below, we over-sample each speckle grain appearing at the output to maintain a good SNR, which leads to a highly rectangular and non-invertible F matrix. Hence, for the general case, we find the modal representation of *E*_*target*_ using the Moore-Penrose pseudo-inverse of F, *F*^+^ as described in Eq. 1.

(1) $$M_{\text{target}\;}=F^{+}E_{\text{target}\;}$$

Here, *M*_*target*_ is *N*_*m*_ × 1 vector of mode coefficients corresponding to the vectorized 2D field *E*_*target*_ , *F* is the *N*_*out*_ *× N*_*m*_ mode matrix and *F*^+^ denotes its pseudo-inverse. In order to tune the mode composition of the target field, we find the orthogonal projection of *E*_*target*_, $E_{\text{target}\;}^{\prime }$ onto a selected subset of $N_{m}^{\prime }<N_{m}$ modes stored in an $N_{\text{out}}\times N_{m}^{\prime }$ mode matrix, *F*′ as shown in Eq. 2.

(2) $$E_{\text{target}\;}^{\prime }=F\prime F\prime^{+}E_{\text{target}}$$

where *F*′^+^ denotes the pseudoinverse of *F*′ defined as *F*′^+^ = (*F*′^†^*F*′)^−1^*F*′^†^ and † denotes the conjugate transpose. In physical terms, $E_{\text{target}\;}^{\prime }$ is the closest output field (least norm solution of the least squares problem) to *Etarget* that can be generated with the selected *Nm*′ modes.

The measured complex-valued TM of the fiber, *K*_*obs*_ is then used to generate the mode tailored field $E_{\text{target}\;}^{\prime }$ at the fiber distal end by projecting an optimal phase mask on the fiber proximal end, *E*_*in*_ calculated using Eq. 3.

(3) $$E_{in}=K_{obs}^{†}E_{\text{target}\;}^{\prime }$$

**Fig. 2.**
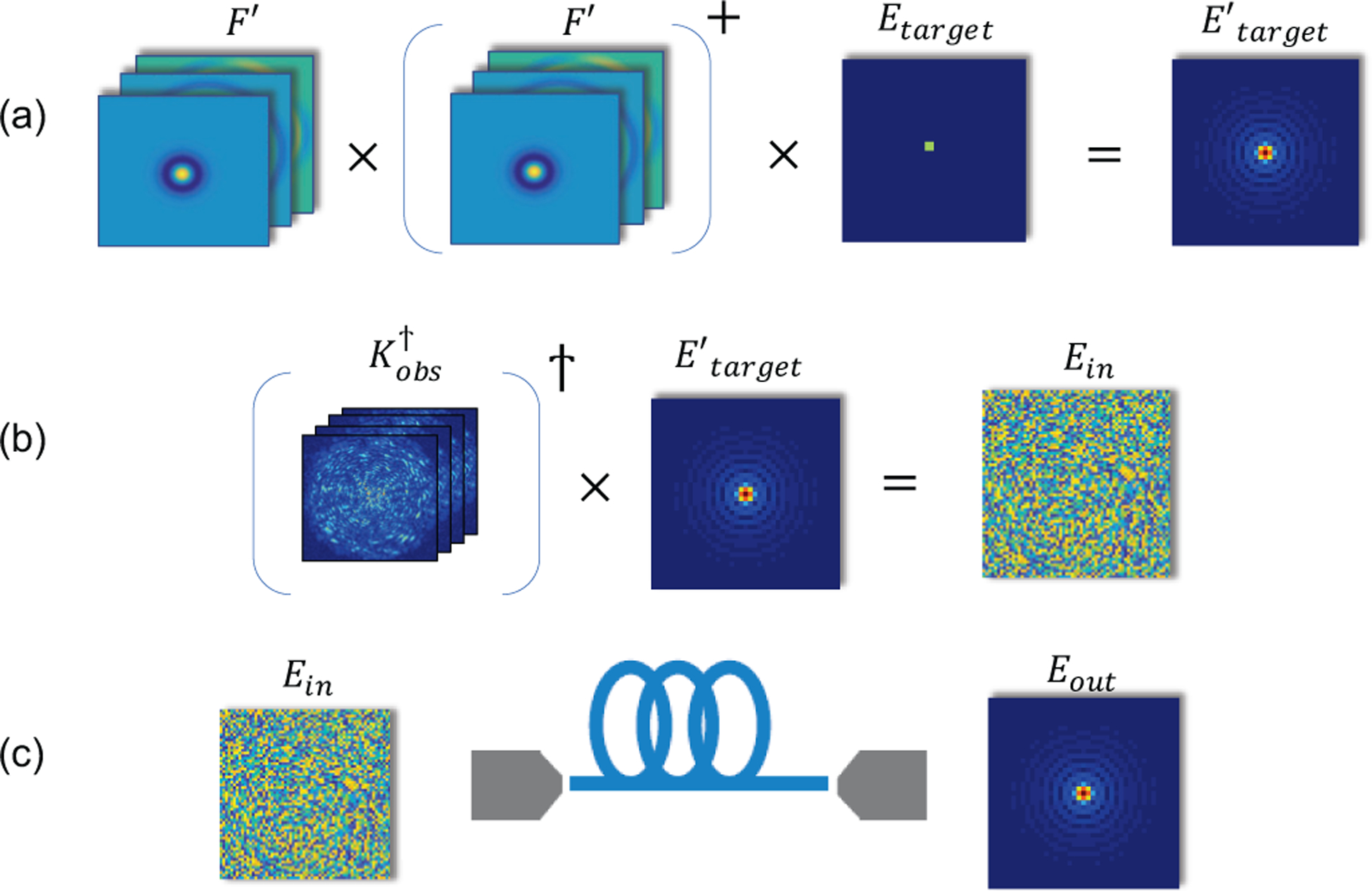
Illustration of mode control for focusing through a fiber using its transmission matrix. (a) The projection of the target field onto the selected mode subset yields its mode-tailored approximation, $E_{\text{target}\;}^{\prime }$. (b) The optimal input phase mask, *E*_*in*_ required to produce the target field at the fiber distal end is found using the conjugate transpose of the transmission matrix. (c) The optimal mask is projected on the fiber proximal end, to produce the output field $E_{out\;}=E_{\text{target}\;}^{\prime }$ after propagation through the fiber. The space dimension in all variables is extended from 1D to 2D only for visualization.

Fig. 2 depicts the entire process with all the olumns reshaped to 2D only for visualization.

We define two metrics to evaluate the mode control performance at the output fields, efficiency and fidelity. The efficiency, denoted by *η*, is defined as the ratio of total energy in the selected fiber modes and the sum total energy in all the modes as shown in Eq. 4.

(4) $$\eta \left(E_{out\;}\right)=\frac{{\left\|E_{out\;}^{\prime }\right\|}^{2}}{{\left\|E_{out\;}^{\prime }\right\|}^{2}+{\left\|E_{out\;}^{\prime \prime }\right\|}^{2}}$$

In the equation above, the single and double primes denote the selected and non-selected mode components of the experimental output field *E*_*out*_, which were calculated by back-projecting the output field, *E*_*out*_ on the fiber modes basis by multiplying it with *F*′*F*′^+^ and *F*″*F*″^+^ respectively. The fidelity on the other hand, denoted by *C*, is defined as the Pearson correlation coefficient between the target and experimentally obtained intensities as defined in Eq. 5.

(5) $$C\left(I_{\text{out}},I_{\text{target}}\right)=\frac{COV\left(I_{\text{out}},I_{\text{target}}\right)}{\sigma_{I_{\text{out}}}\sigma_{I_{\text{target}}}}$$

Here, COV denotes the covariance function and *σ* denoted the standard deviation of the variable in the subscript. While the efficiency characterizes the confinement of energy in the selected modes, the fidelity characterizes the spatial control ability.

## III. Experimental setup

**Fig. 3.**
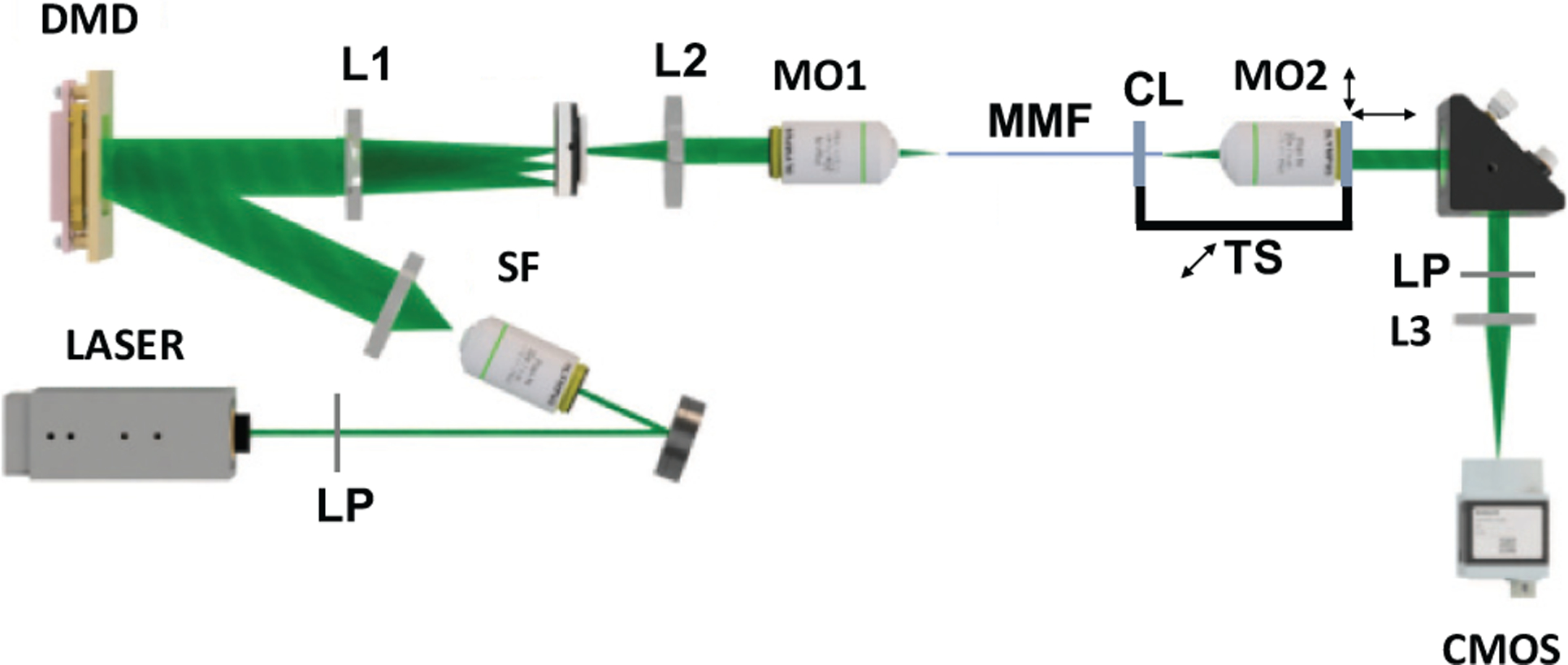
Experimental setup for mode control and focusing through an MyF. L1, L2, L3: lenses, MO1, MO2: Microscope objectives for coupling light in and out of the fiber, LP: Linear polarizer. SF: Spatial Filter, CL: Clamp to hold to fiber distal end, TS: 1D translation stage used for bending the fiber, CMOS: Camera to measure the distal end intensity.

The experimental setup used in our experiments is illustrated in Fig. 3. It consists of a 532 nm, CW laser and a binary amplitude digital micromirror device (DMD) that can be used for phase modulation using computer-generated holography. Holography enables recording of arbitrary wavefronts with binary-amplitude modulation [44], [45]. DMDs have an advantage over liquid crystal SLMs due to their orders of magnitude faster refresh rate and polarization insensitivity.

Because the DMD provides only binary-amplitude modulation, the effective number of phase pixels (4096 in our case) is significantly lower than the number of binary pixels (262144). A microscope objective couples the Fourier transform of the modulated wavefront into the MyF and another microscope objective and lens L3 are used to image the MyF distal tip onto a CMOS camera. We use a ~ 40 cm long graded index MyF with a diameter of 100 *μ*m (Newport F-MLD) for all experiments. We place a linear polarizer at the distal tip to limit the TM measurement to a single polarization. In the absence of polarization coupling, this would mean that the 4096 independent phase pixels of the DMD control 3570 single polarization modes of the MyF. However graded index fibers show significant polarization coupling [46] which leads to loss of some light to the unoptimized orthogonal polarization and in turn reduces the focus enhancement, defined as the ratio of the peak focal intensity and the average output intensity. In any case, extension of the approach to two polarizations in the TM is relatively straightforward [47], [48].

Different sets of basis functions can be chosen to measure the TM, including canonical plane waves or focal spots at the input facet of the fiber. For this study, we chose the Hadamard phase basis because of its ease of implementation with the DMD. The calibration required 12288 measurements which were made in about three minutes. The calculation of the pseudoinverse of the mode matrix has a complexity of $O\left(N_{in}N_{out\;}^{2}\right)$ and is made in advance to determine the mode selected $E_{\text{target}\;}^{\prime }$ fields. After calibration,we used the TM to generate phase conjugated focal spots at the output. We choose focal spots because of their importance in imaging. In addition, they are easily generated using the conjugate transpose approximation of the inverse of the TM. Generation of more complex patterns is also possible, although it requires a regularized TM inversion which is computationally more demanding [49]. The optimal phase masks to project each focal spot are determined using Eq. 3 with a computational complexity of *O*(*N*_*in*_*N*_*out*_). Each generated focal spot fields, *E*_*out*_ was measured using three phase measurements, just as done during calibration and their corresponding mode coefficient vectors, *M*_*out*_ are determined using Eq. 1, but for *E*_*out*_ instead of *E*_*target*_.

## IV. Results

**Fig. 4.**
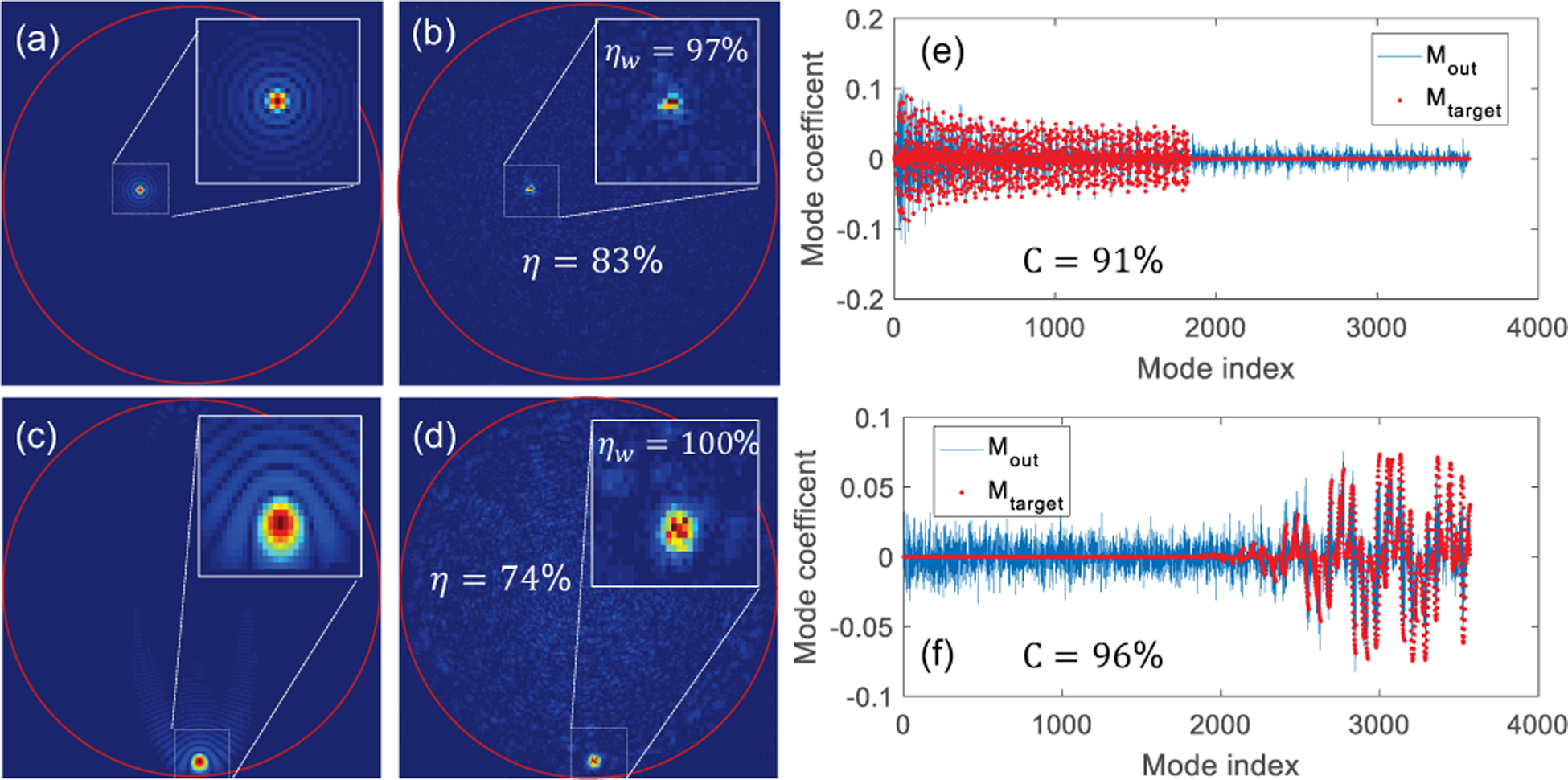
Experimental demonstration of focusing with mode control: (a,b) Absolute value of the expected and experimental electric fields respectively when focusing using LOMs. (c,d) Absolute value of the expected and experimental electric fields respectively, when focusing using HOMs. (e) Modal composition of fields in (a) and (b). (f) Modal composition of fields in (c) and (d). Insets display a zoom-in on the focus profile. Experimental efficiencies of full fields, *η*, and of cropped windows, *η*_*w*_, are indicated in respective figures and the fidelities, C, are indicated within their mode coefficient plots.

To demonstrate mode tunability, we created focal spots using two subsets of modes in the mode group-ordered mode matrix *F*. A first set of scanning spots was created with the half lowest order modes (LOMs) in F and the second set of spots was created using the half highest order modes (HOMs). Figure 4 illustrates two examples of focal spots, one using each of the above two mode sets. Figure 4 (a–d) show the absolute value of the target and experimental output fields, $E_{\text{target}\;}^{\prime }$ and *E*_*out*_ for the two focal spots and figure 4 (e,f) show their corresponding targeted and experimental mode compositions, $E_{\text{target}\;}^{\prime }$ and *M*_*out*_. We can observe that while the mode coefficients of the non-selected modes cannot be completely suppressed in the experiment, the mode coefficients of the selected modes are in good agreement with their targeted values.

The high efficiency and fidelity values obtained for the focal spots generated with selected modes are indicated in their respective sub-figures in Fig. 4. We also analyzed a circular window of radius 8 pixel wide around the focal spot for both fields and the corresponding efficiency values are shown in the top-right zoom-in insets. The increase in efficiency indicates that although some energy remains in the unselected modes within the full field, the focal spot is primarily a result of the interaction of the selected modes.

**Fig. 5.**
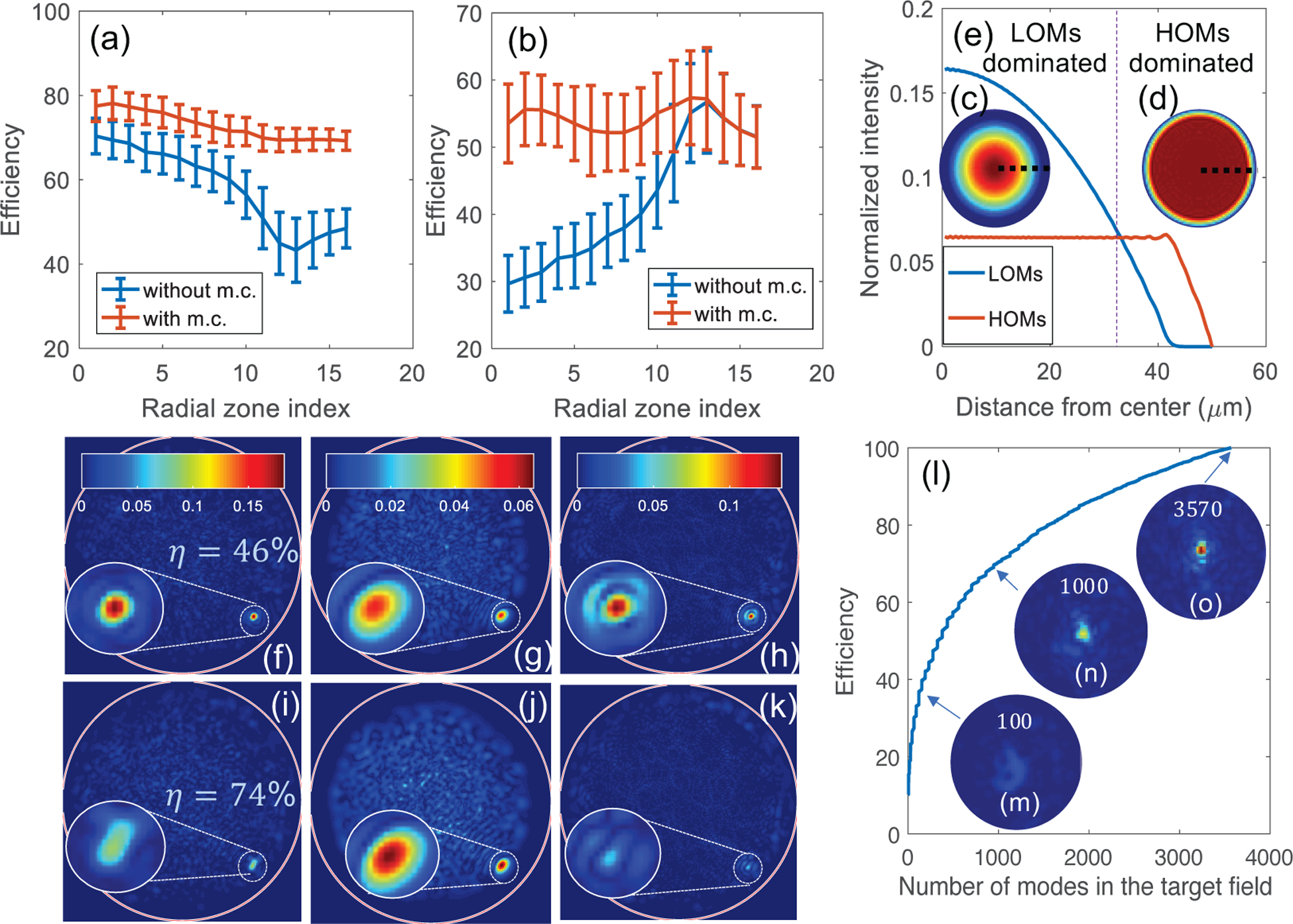
Statistics of mode control through MyFs as a function of radial location of the focal spots and the number of modes selected for optimization. All sub-figures show data simulated using the experimentally measured TM. (a,b) Average efficiency with standard deviation errorbars of focal spots as a function of their radial location (1965 focal spots). The focal spots are evenly spaced across the entire fiber cross section and split into 16 radial zones for the plot. We define radial zones as equal area annuli/circle with increasing inner and outer radii. The number of focal spots in each radial zone is 121 ± 7. (a) Comparison of LOM efficiencies for focal spots created with mode control using only LOMs (red curve) and without mode control or using all the fiber modes (blue curve). (b) Comparison of HOM efficiencies for focal spots created with mode control using only HOMs (red curve) and without mode control or using all the fiber modes (blue curve). (c, d) Net radial intensity profile of (c) LOMs and (d) HOMs mode sets. (e) Cross section of the LOMs and HOMs profiles corresponding to the thick dotted lines marked in (c) and (d). A thin dotted line divides the fiber cross section into “LOMs dominated” and “HOMs dominated” regions, depending on which mode set has a higher intensity profile in the region. (f-k) Example of a digitally computed focal spot (f,i) and their corresponding LOMs (g,j) and HOMs (h,k) components created using all the fiber modes or without mode control (f-h) and using LOMs or with mode control(i-k). (f) Digitally computed focal spot created without mode control. (g) LOMs component of the focal spot field in (f) computed by back projecting the field on the fiber mode basis as described in the main text. (h) HOMs component of the focal spot field in (f). (i) Focal spot field at the same location as in (f) but digitally computed using only LOMs outside its optimal region. (j) LOMs component of focal spot field in (i). (k) HOMs component of focal spot field in (i). The proportion of energy in the LOMs in the spot fields shown in (f) and (i) are indicated inside the figures. (l) Efficiency of the focal spot shown in Fig. 4 (b), digitally computed using, different number of mode coefficients from its full mode coefficient set, *M*_*target*_. (m-o) Zoom-ins of the evolving focal spot when 100, 1000 and all the 3570 modes are considered in the target field.

Interestingly, when focusing in the near field of the distal end of the fiber, the mode efficiency varies with the radial location of the focal spot when a given HOM or LOM set is selected. This is because different modes have different spatial support and are more or less suitable for the target output. To demonstrate this, we show plots of efficiency of mode-controlled focal spots created at increasing distance from the center of the fiber using LOMs and HOMs respectively [Fig. 5 (a) and (b) (red curves)]. The plots represent the statistics of 1965 focal spots spread evenly across the fiber cross section. All focal spot fields are digitally computed using the experimentally measured TM and the optimal phase mask calculated in Eq. 3. We observe that when focusing with LOMs, the efficiency decreases away from the center of the fiber and flattens at the boundary, while for the HOMs, the efficiency decreases between radial zones 5 – 10, increases near the boundary and then decreases again. Both these trends roughly follow the net intensity profiles of the LOMs and HOMs mode sets respectively, which are shown in the circular insets of Fig. 5 (e) [Fig. 5 (c) and (d)]. The intensity profiles are calculated as the sum total intensity of all the modes in a mode set. The cross sections of the intensity profiles of the two mode sets are plotted in Fig. 5 (e) and show that the LOMs dominate the central fiber region and do not extend all the way to the boundary, where the HOMs start to dominate. Hence, focusing in the central region is optimal for mode control using LOMs, while focusing in the outer boundary region is better done with HOMs. Even without mode control, these choices intrinsically yield the highest efficiency values.

To compare the optimized mode compositions of focal spots with their corresponding intrinsic ones, we also show in Fig. 5 (a) and (b), the efficiency of LOMs and HOMs respectively for the focal spots created without mode control or using all the fiber mode (blue curves). When we do not employ mode control, we calculate the efficiency as the intrinsic proportion of energy in the particular mode subsets chosen in Fig. 5 (a) and (b). It can be noted that even as the focus moves away from the regions where the selected mode sets dominate, which we refer to as their corresponding optimal regions, mode control enables creating focal spots with up to 66% and 73% more energy in the selected modes. The only exception is observed at the boundary of the fiber when HOMs are selected. For this special case, the mode control makes no difference in the efficiency, which makes sense since LOMs do not extend till the fiber boundary and hence cannot contribute to focal spots at the boundary.

In general, although the efficiency and fidelity values can be lower outside the optimal region of mode sets, the proportion of energy in the selected modes improves significantly due to mode control. To visualize this improvement, we show the digitally computed output fields and their LOM and HOM components for a focal spot created without and with mode control i.e., using all the fiber modes and using LOMs [Fig. 5 (f–h) and (i–k) respectively]. Mathematically, the output fields, *E*_*out*_ [5 (f) and (i)] were computed as the product of the input fields *E*_*in*_ found in Eq. 3 with *K*_*obs*_. The $E_{\text{target}\;}^{\prime }$ used to calculate the input fields were calculated using all the fiber modes i.e., $E_{\text{target}\;}^{\prime }=E_{\text{target}}$ for 5 (f) (without mode control) and using Eq. 2 with *F*′ containing only LOMs for 5 (i) (with mode control). The LOMs and HOMs mode components of each of the above output fields [5(g,h) and 5(j,k)] were found by back-projecting the output fields, *E*_*out*_ on the fiber modes basis by multiplying them with *F*′*F*′^+^, where *F* is the fiber mode matrix with the LOMs or HOMs in its columns. The HOMs component is non-zero even when LOMs are chosen to create the spot field because the mode control efficiency is not 100 %.

The particular spot shown is created near, but not quite at the fiber boundary. We find that even for LOM selection, mode control allows improving the proportion of LOM energy from 46% to 74% and suppresses the energy in the HOMs. Furthermore, the contribution from LOMs to the focal spot alone is also enhanced from 28% to 78%, while the contribution from HOMs is diminished to 22%. It should be noted, that although mode control succeeds in putting more energy in LOMs even when the focal spot is created outside their optimal regions, the enhancement decreases. This is explained by the fact that the non-selected HOMs, which dominate the region, no longer participate in forming the focal spot when mode control is employed. Appendix B, Fig. 7 shows experimental examples of two focal spot fields, and their corresponding mode coefficients, created outside their optimal regions.

Another interesting aspect of this method is that it is more efficient in generating mode-controlled fields that involve interaction of many modes rather than few modes or just a pure mode. This can be explained by the fact that as the number of modes interacting in the target field increases, the higher the number of optimized modes is and the weaker the unoptimized speckle background becomes. Fig. 5(l) illustrates this phenomenon. Each data point represents a unique mode control optimization and as the number of modes in the target field increases, we observe an increase in the efficiency of the mode-controlled output field. The output fields at all points are digitally computed from the TM and the target field as before. For this plot, we chose the mode coefficients of the focal spot shown in Fig. 4 b, and performed different optimizations using its 1st, 1st and 2nd, 1st 2nd and 3rd, and so on with the following cumulative mode coefficients. We normalized all the target fields by their Euclidean norm for calculating the efficiency. Without this normalization, the evolution of the target and output fields at each data point can be visualized in the accompanying movie, Visualization1.mp4. The insets in the plot in Fig. 5 (l) labelled 5 (m-o) show zoom-ins of the evolving output focal spot when the first 100, 1000 and all the 3570 mode coefficients respectively are considered in the target field. We can observe that as more modes are selected, the unoptimized background due to the unselected modes decreases, leading to better efficiency as well as focus enhancement.

**Fig. 6.**
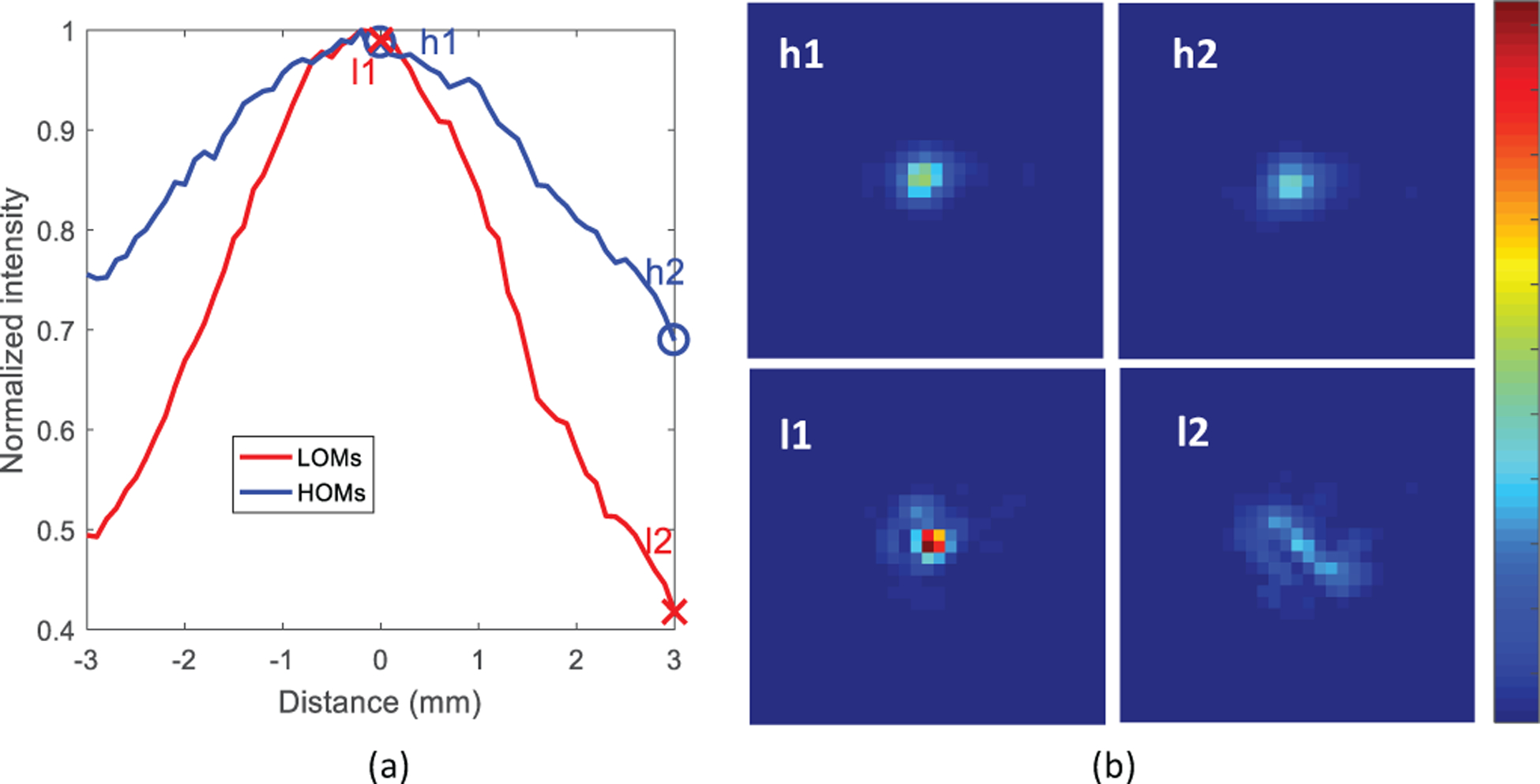
Experimental demonstration of bending resilience of focal spots created using different mode sets. (a) Plot of normalized peak focal intensity of 200 near-axis focal spots created using LOMs(red) and 200 near-boundary focal spots created using HOMs (blue) with translation of the fiber distal tip. (b) Evolution of example focal spots l1 and h1 created using LOMs and HOMs respectively at the initial fiber position into l2 and h2 after a 3mm translation of the distal tip. The positions of the spots are marked in (a).

**Fig. 7.**
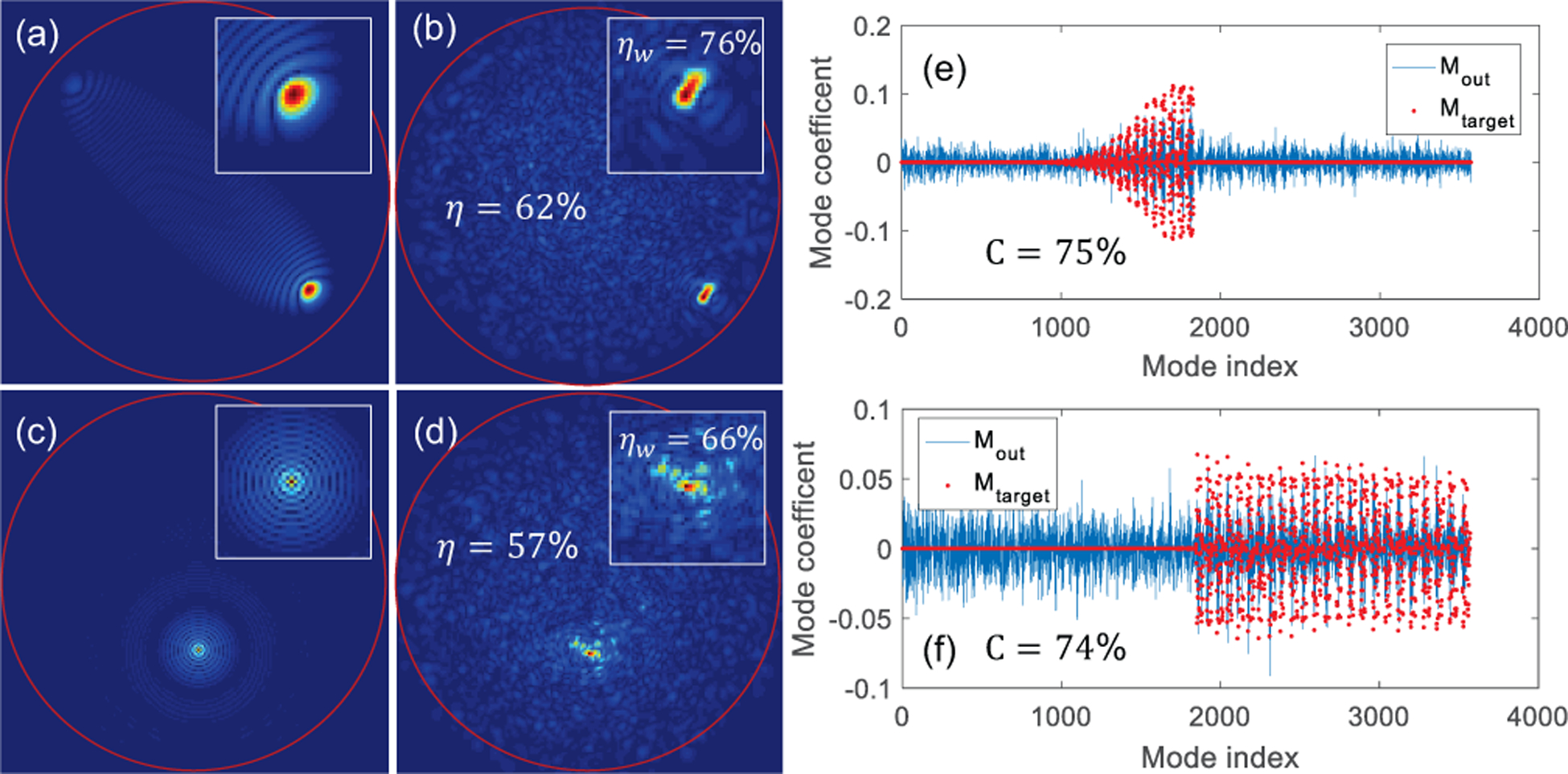
Experimental demonstration of focusing with mode control outside the optimal regions of the respective mode sets: (a,b) Absolute value of the expected and experimental fields respectively when focusing using LOMs. (c,d) Absolute value of the expected and experimental fields respectively, when focusing using HOMs. (e) Modal composition of fields in (a) and (b). (f) Modal composition of fields in (c) and (d). Insets display a zoom-in on the focus profile. Experimental efficiencies of full fields, *η*, and of cropped windows, *η*_*w*_, are indicated in respective figures and the fidelities are indicated along with their mode coefficient plots.

Finally, using inferences from Fig. 5 about the mode efficiencies of focal spots at various locations, we studied the robustness of different mode-controlled focal spots to fiber bending. Towards this end, we generated 200 focal spots, each using either LOMs or HOMs in their optimal regions (near fiber axis and at the boundary respectively). To test the robustness, we mount together the fiber clamp, CL and the objective MO1 shown in Fig. 3 on a translation stage in order to introduce controlled movements to the fiber distal tip along the horizontal axis. The intensities of the focal spots are recorded in displacement steps of 100 *μ*m, upto 3 mm. Fig. 6 (a) shows the change in the peak intensity averaged over 200 focal spots with translation and Fig. 6(b) shows the evolution of two example focal spots from each of the two mode sets over the motion range. It can be observed that the spots formed with HOMs retain a 30% higher focal intensity than those formed with LOMs after a translation of 3 mm. This indicates that focal spots created using HOMs in the boundary are more robust under these experimental perturbations. This improved robustness could be a result of reduced intermodal coupling and the better stability of high orbital angular momentum modes [10], [50], [51]. Interestingly, the insight from Fig. 5 (a–e) about the lack of participation of LOMs in focal spots created at the fiber boundary supports this explanation.

## V. Discussion and conclusion

We have demonstrated a method to select the mode composition at the output of a myriad-mode fiber (MyF). While we create focal spots at the fiber output, the technique can be extended to generate any desired complex output fields within the limits of the physical mode content of the fiber. We have shown that mode selection with considerable accuracy is possible when the focal spot is created at a proper output position in the fiber cross-section. A key aspect in our experiments is that the number of independent fiber modes per polarization was comparable to the number of controllable phase pixels (87%) unlike in prior SLM-based mode control methods that use thousands of pixels for fibers with about 100 – 200 modes.

The efficiency and fidelity figures demonstrated here could be improved, for example, by enabling simultaneous amplitude and phase modulation, including full polarization control, and by employing adaptive alignment techniques to enhance mode overlap and coupling efficiency [29], [52]. Furthermore, we did not take any special measures for thermal or mechanical stabilization in our experiment, so our results could be affected by any perturbations to the fiber after the TM calibration. Moreover, we used a 40 cm long fiber prone to misalignment, bends and intermodal and polarization coupling. Using shorter fibers can greatly reduce the magnitude of all these effects and lead to closer to LG-like mode profiles [53]. Additionally, while the LG modes are a good model for graded index fiber, it is well known that commercial fibers have less than perfect index profiles. More precise estimates of the true modes can be attained by a singular value decomposition of the fiber’s TM or using other mode characterization techniques [52], [53], [54]. Mode control performance is also limited by imperfections arising from the wavefront shaper. Phase errors can occur from the imperfect phase encoding of the binary amplitude holograms employed for phase modulation with the DMD. Another source of phase error is the 8-level discretization of the phase patterns projected on the DMD. The coupling efficiency of the phase pattern projected from the DMD into the fiber is also a critical factor in mode controllability. For instance, the coupling efficiency of the higher order Hadamard functions to the fiber is poor and can restrict the controllable fiber modes, suggesting other bases might provide even better performance.

As opposed to prior work, efficient mode control in MyF is much more challenging due to the limited number of degrees of freedom provided by the DMD and the inherent complexity of the system. However, the approach ensures that maximum energy is confined to the selected modes at the output and although all the fiber modes still propagate to the distal end of the fiber, the focal spot itself, which is many times brighter than the background, is primarily a result of the interaction of the selected modes. It should also be emphasized, that the technique controls the mode composition at the output of the fiber, which is not necessarily the same as the mode composition throughout propagation due to mode coupling resulting from perturbations of the fiber (bending, imperfections, etc). Because the mode composition throughout the fiber is complex, our technique is more suitable for generating complex mode combinations rather than a combination of fewer modes or a pure mode, unlike other traditional mode control techniques.

The proposed method also provides an avenue for combining the advantage of large core MyFs for a larger bandwidth, higher NA and bigger field of view, with the bend resilience of an MMF with fewer modes. The extent of intermodal coupling or bend sensitivity of an MMF is inversely proportional to the difference between the propagation constants of the modes [55]. Hence, a fiber of a given NA with a small number of modes exhibits better resilience to bending than one with a larger number of modes. By only selecting a subset of modes while shaping the output wavefront of an MyF, we can ensure reduced intermodal coupling and hence improve the fiber’s robustness. Here, we performed an experiment to observe the bend resilience of different mode sets and found that focal spots created using HOMs in the boundary of the fiber show improved resilience. Interestingly, better robustness of HOMs, high frequency speckles composed of HOMs, and/or near-boundary spots has also been reported in other types of robustness experiments with MMFs involving translation of s-bends [56] or bending the central part of the MMF [25], [57], [58]. Although we observed better robustness only in the boundary, we can generate arbitrary fields in the far field of the fiber using these bend resilient modes to achieve better robustness overall [59]. These insights could prove helpful in controlling the bend resilience of an MyF for both imaging and communication applications.

The idea of mode selection through few-mode MMFs is already a topic of wide interest in the field of fiber-optic communication. Mode selection in MyF, could allow the use of groups of modes with similar dispersion and delay profiles as different channels, where the number of channels can be smaller than the total number of modes in the fiber. Other possible applications of mode selection in MyF include control of individual mode groups for spatio-temporal focusing, quantum communication, and energy delivery.

## Supplementary Material

supp1-3057615

## Figures and Tables

**TABLE III:** Cross-fidelity and RMSE calculations between digitally computed and experimental output intensities.

Focal spot	*C*_*dig–expt*_ (%)	*RMSE*_*dig–expt*_ (%)
Spot 1 (LOMs)	96.45	22.06
Spot 2 (HOMs)	99.4	10.12

**Fig. 8.**
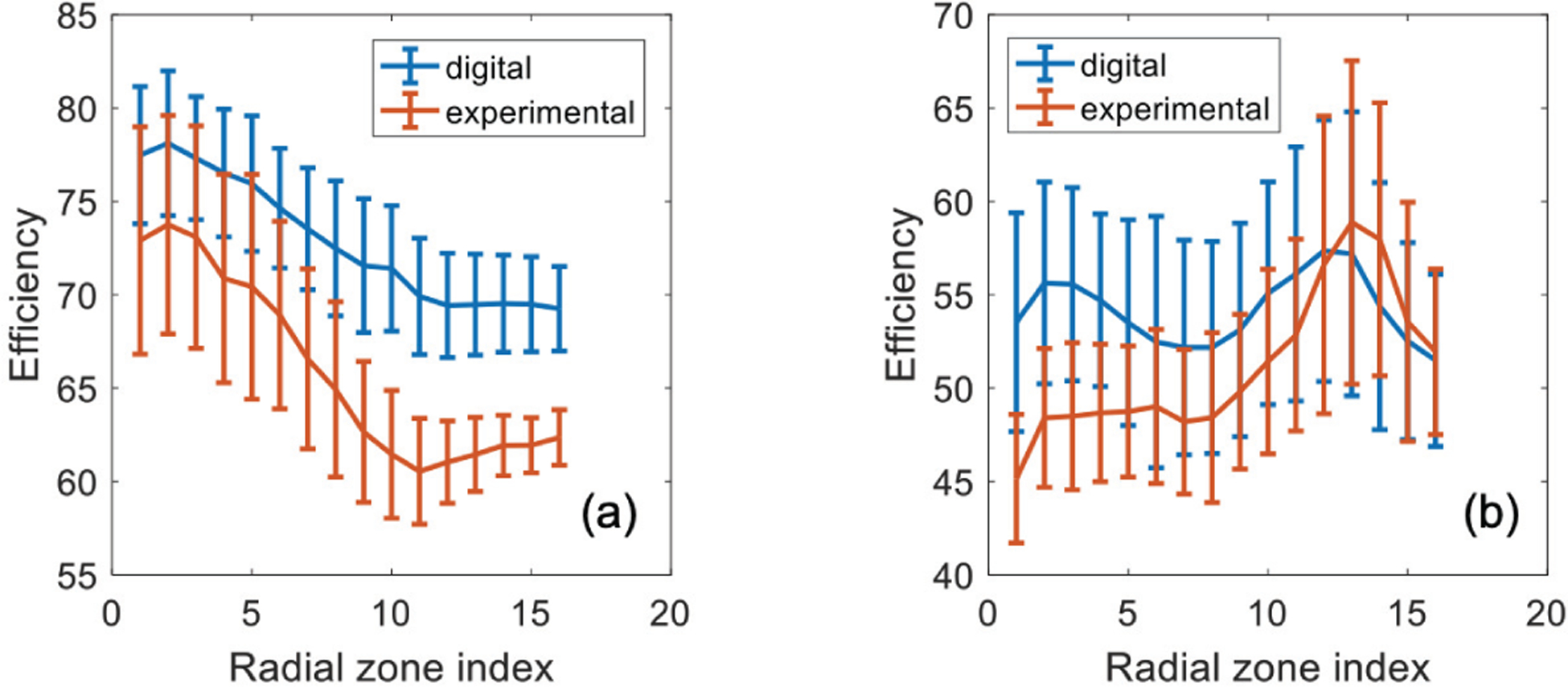
Average efficiency of focal spots as a function of their radial location (1965 focal spots). The error bars denote standard deviation values. Radial zones are equal area annular/circle across the fiber section, as defined in Figure. 4 of the main text, each containing 121 ± 7 focal spots. (a) Comparison of efficiencies w.r.t LOMs for mode-controlled focal spots created digitally (red curve) and experimentally (blue curve). (b) Comparison of efficiencies w.r.t HOMs for mode-controlled focal spots created digitally (red curve) and experimentally (blue curve).

**Fig. 9.**
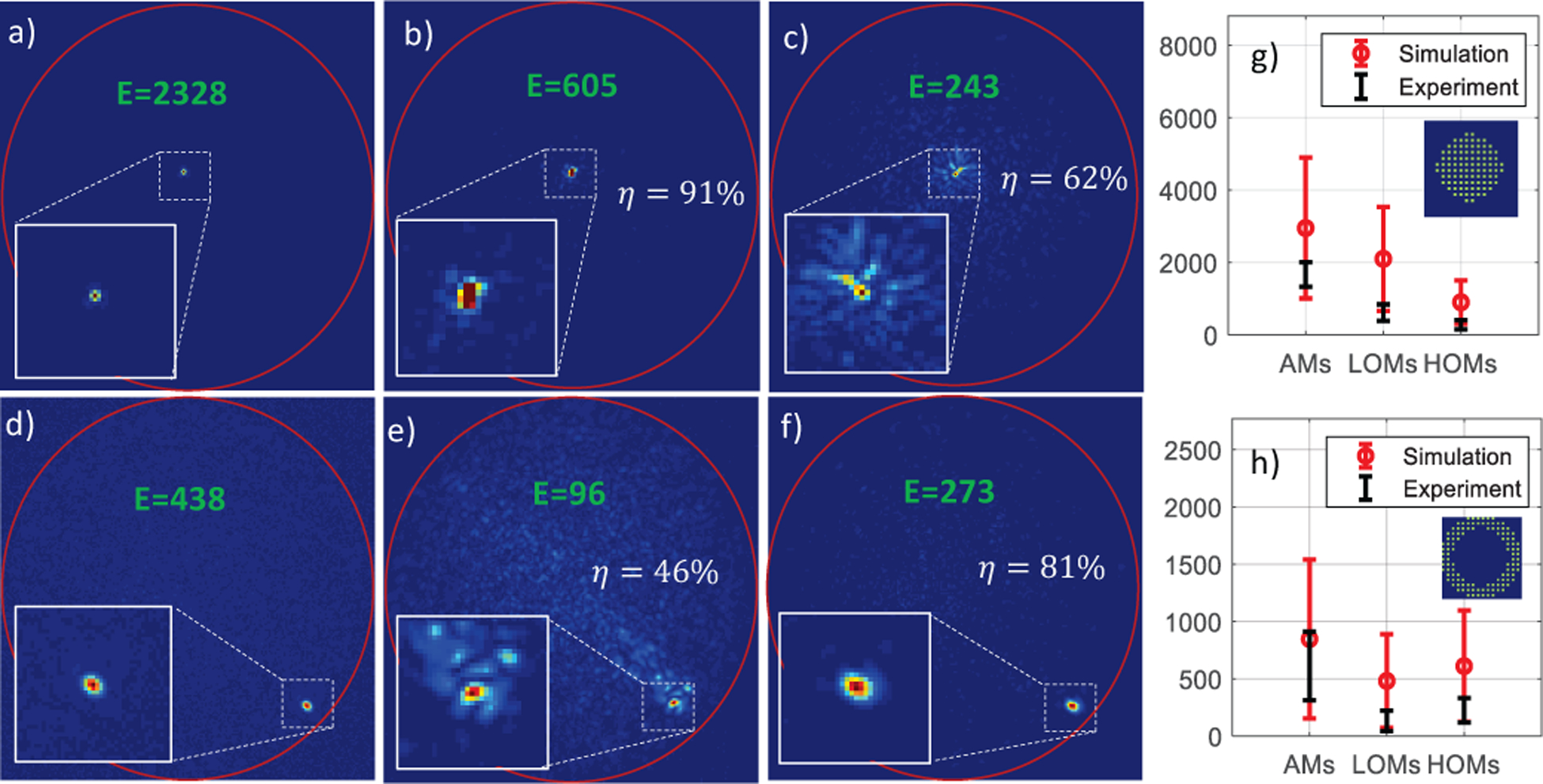
Enhancement as a function of radial position and mode selection. (a) Example of a near-axis focal spot intensity image created without mode control, (b) the same spot created using LOMs, and (c) using HOMs. (d) Example of a near- boundary focal spot intensity image created without mode control, (e) created using LOMs, and (f) using HOMs. The enhancements, E are indicated in green in the respective intensity images and the insets show a zoom-in on the focus profile. The experimental mode efficiency, *η* measured from their respective fields are also shown for the focal spot fields created with mode control. (g) Enhancement statistics of near- axis spots and (h) near-boundary spots. The spots in (g) and (h) belong to a circular inner region and an equal area annular outer region respectively in the fiber cross-section. Their exact locations are indicated in the respective figure insets. The x-axis indicates the set of modes chosen to generate the focal spots, AMs or all modes and LOMs and HOMs respectively. The y-axis shows the focus intensity enhancement. The black error bars correspond to experimental data, while the red error bars denote digitally computed focal spots using the experimental TM.

**Fig. 10.**
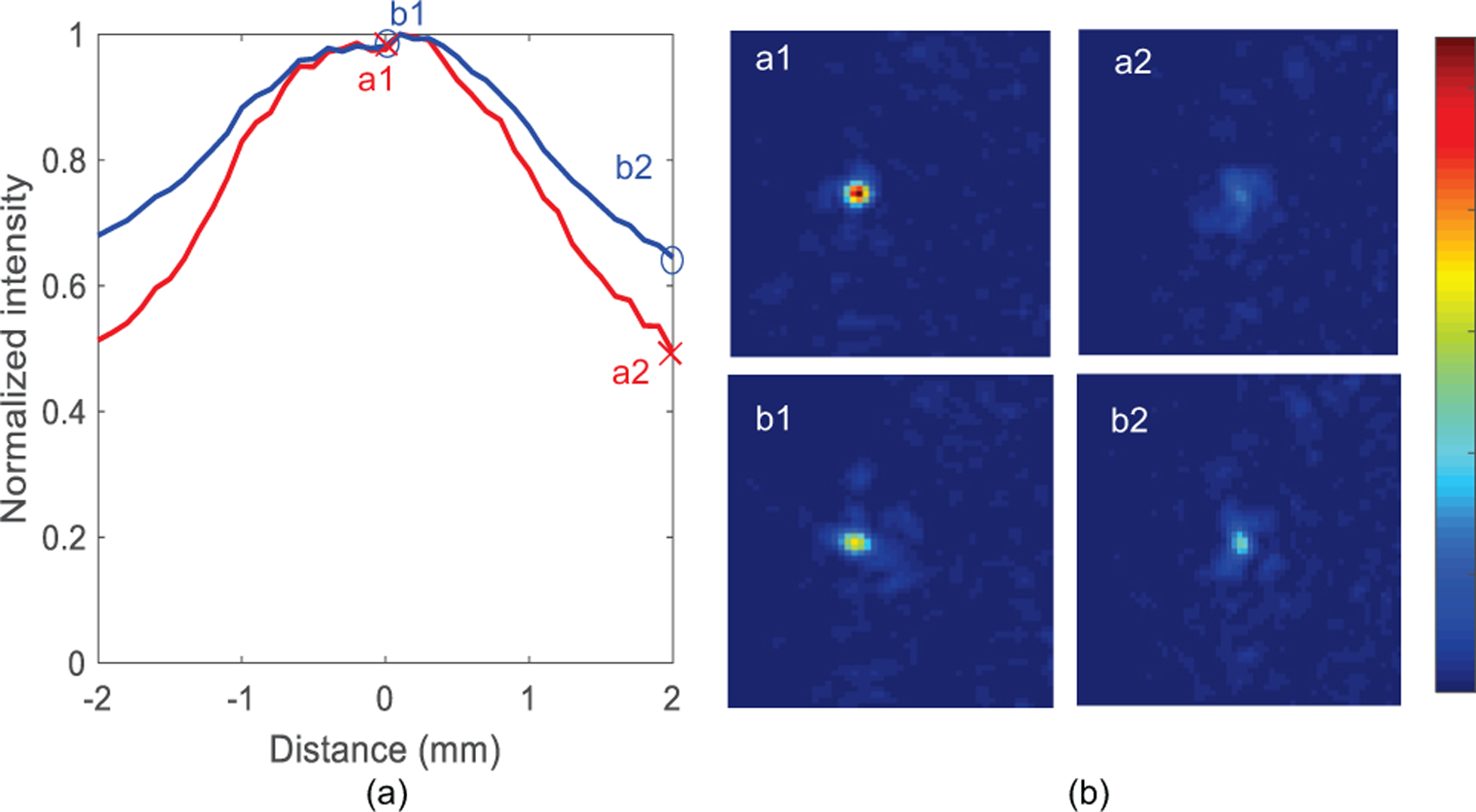
Experimental demonstration of bending resilience of focal spots created with and without mode selection. (a) Plot of normalized peak focal intensity of 150 near-boundary focal spots created using AMs (red) and the same focal spots created using HOMs (blue) with translation of the fiber distal tip. (b) Evolution of representative focal spots a1 and b1 created using AMs and HOMs respectively at the initial fiber position into a2 and b2 after a 2 mm translation of the distal tip. The displacement positions of the spots are marked in (a).

## Appendix A Experimental calibration of the transmission matrix of a myriad mode fiber

The transmission matrix (TM) defines the relationship between the input and output modes of the myriad mode fiber (MyF). A vectorized output $\text{field}E_{out\;}^{m}$ appearing on the distal side of the fiber, where *m* is the output mode index, can be described as a weighted sum over all the vectorized input modes stored in a matrix, *E*_*in*_ launched into the fiber, each with a weight *t*_*mn*_ corresponding to the *m*^*th*^ output mode and *n*^*th*^ input mode, as described in Eq. 6.

(6) $$E_{out\;}^{m}=\overset{N}{\underset{i=1}{\sum }}t_{mn}E_{in\;}^{n}$$

The set of TM coefficients for all N input modes and M output modes generates the full TM. In order to measure these weights experimentally including both phase and amplitude information, we project a complete basis set of orthogonal input fields into the fiber proximal tip accompanied with a phase-stepping reference field. In most cases these fields are constant in amplitude with their phase dynamically modulated. If using an amplitude spatial light modulator such as the DMD, phase modulation is achieved by projecting computer-generated amplitude holograms [44]. The DMD’s active area is divided into two sections, one (typically centered) for the basis-function changing pattern and another for the phase-stepping reference, typically surrounding the first one. The intensity measurements of the fiber output for each projected pattern, as the reference field is phase stepped three times, enables recovery of the output field using phase-shifting interferometry as described in Eq. 7.

(7) $$t_{*n}=\frac{I_{n}^{0}-I_{n}^{\pi }}{4}-i\frac{I_{n}^{0}-2I_{n}^{\pi /2}+I_{n}^{\pi }}{4}$$

Here $I_{n}^{i}$ denote the output mode intensities for the n^th^ input mode and their superscripts denote the phase step of the reference field. Repeating the above output field measurements for N input modes gives us the observed TM, which we denote as *K*_*obs*_. *K*_*obs*_ is only an estimate of the fiber TM, since the reference field employed for calibration propagates through the fiber and transforms into a speckle pattern instead of an ideal plane wave reference typically employed for interferometry.

## Appendix B Focusing with mode control outside the optimal region of a mode set

As shown in Fig. 5 of the main text, if we create a mode controlled focal spot in the non-optimal region of the selected mode set (HOM or LOM), we can direct significant energy from the dominant modes in the region to the non-dominant selected modes. We have defined the optimal region for a mode set as the spatial region where the selected mode set contains more net energy relative to its complementary mode set and vice versa for the non-optimal region. Here we show the experimental version of the digitally computed focal spot shown in Fig. 5 (i), where we focus near the fiber boundary using lower order modes (LOMs) [Fig. 7 (a, b)]. We also show a second focal spot created near the fiber center using HOMs in Fig. 7 (c, d). The corresponding target and experimental mode coefficients are also shown in Fig. 7 (e, f). We observe that the efficiencies and fidelities are lower for these examples, however the proportion of energy in the selected modes due to the mode control optimization is still significant.

## Appendix C Comparison of efficiency statistics of experimental and digitally computed mode-controlled output fields

Here we compare the mode control efficiencies of experimentally generated focal spots with those computed digitally using the measured TM. The efficiency plots when focusing using LOMs and HOMs are shown in Fig. 8 (a) and (b) respectively. We observe that the trend of the two curves matches quite well. The experimental values are in general lower, most likely due to measurement noise, perturbations to the fiber and phase errors from the DMD.

## Appendix D Enhancement statistics of experimental and computed mode-controlled output fields

The enhancement of a focal spot, defined as the ratio of peak focal intensity and the average image intensity, also varies with the radial position. In Fig. 9 we show two focal spots- one near the fiber axis and another near its boundary, each created using all the fiber modes (AMs), LOMs or HOMs. The all-mode data corresponds to focal spots created without mode control. We observe for both spots, as expected, that the best enhancement is achieved when all the modes are optimized and least is achieved when we use the non-dominant modes to create the focus. Focal spots created in the optimal regions of the selected mode sets yield intermediate enhancement, but the best mode efficiencies. The error bars for the enhancement statistics also confirm the above observation, and the trend of the enhancement with varying mode compositions matches well for the digitally computed or simulated focal spots and experimental focal spots. However, the standard deviation of the errorbars of the simulated focal spots is larger than that of the experimental focal sports. While this is not straightforward to explain, we point to the fact that the simulated enhancement statistics shown in Fig. 9 is based on the experimentally measured transmission matrix, which is also prone to measurement noise. Finally, we observe that the maximum achievable enhancement for near-boundary spots are smaller but remain steadier with varying mode compositions than the near-axis spots.

## Appendix E Influence of mode selection on robustness

We compared focal spots created with and without mode selection to observe the effect of using fewer modes and mode selection on robustness. We generated 150 focal spots, each with all the modes (no mode selection, AM) and with HOM selection. The focal spots were created in the same positions for both mode selections unlike in the comparison shown in Fig. 5 of the main text. We moved the MyF distal tip along the horizontal axis in steps of 100 *μm*, up to 2 mm. Fig. 10 (a) shows the change in the peak intensity averaged over 150 focal spots with translation while Fig. 10(b) shows the evolution of two focal spots over the motion range in both cases. We observe that the spots created using fewer HOMs are ~ 10% more robust than those created using all the modes. This is explained by the fact that when all the fiber modes are used for focusing, the change in the focal spot intensity due to fiber motion is larger as a result of all the modes contributing to the spot. On the other hand, when only the HOMs are used for focusing, the change in the focal spot intensity is smaller because it is a result of fewer mode interactions and lower probability of mode coupling. Although the total change in the output field is the same in both cases, the unselected LOMs in the second case primarily contribute and lead to change in the background intensity.

## Appendix F Mode control metrics

We introduced the efficiency and fidelity metrics in the main text of this paper to evaluate the mode control performance. Various other metrics have been considered. For instance, the same calculations can be done for the digitally computed fields. Furthermore, we can also compare the same focal spots generated digitally and experimentally. In this section, we present additional calculations to make all the above comparisons. All calculations are made for the focal spots in Fig. 4 (b) and (d) of the main text, which are named Spot 1 and Spot 2 respectively.

We generalize the efficiency and fidelity definitions from the main text and denote the efficiency for any given quantity, E as *η*(*E*) and the Pearson correlation between two quantities A and B as C(A,B), which is mathematically expressed in Eq. 8. We also measure the relative mean square error between two given intensity images, defined in Eq. 9.

(8) $$C(A,B)=\frac{COV(A,B)}{\sigma_{A}\sigma_{B}}=\frac{\sum_{i=1}^{N}\left(A^{i}-\bar{A}\right)\left(B^{i}-\bar{B}\right)}{\sqrt{\sum_{i=1}^{N}{\left(A^{i}-\bar{A}\right)}^{2}}\sqrt{\sum_{i=1}^{N}{\left(B^{i}-\bar{B}\right)}^{2}}}$$

(9) $$\text{RMSE}(I2/I1)=\frac{\sum_{i=1}^{N_{out}}{\left(I_{2}^{i}-I_{1}^{i}\right)}^{2}}{\|I_{1}\|{}^{2}}$$

### A. Comparison of experimental target fields and intensities

The efficiency, *η*_*expt*_ = *η*(*E*_*out*_) for the experimental fields, *E*_*out*_ w.r.t. the target field, *E*_*target*_, and the fidelity, *C*_*expt*_ = *C*(*I*_*out*_*, I*_*target*_) and *RMSE*_*expt*_ = *RMSE*(*I*_*out*_*/I*_*target*_) for the experimental intensities, *I*_*out*_ w.r.t the target intensities, *Itarget* , are summarized in Table I. The high root mean square errors can be attributed to the background speckle intensity from the unselected modes.

**TABLE I:** Efficiency calculations for the experimental fields w.r.t to the target fields and fidelity and RMSE calculations for experimental intensities w.r.t the target intensities.

Focal spot	η_*expt*_ (%)	*C*_*expt*_ (%)	*RMSE*_*expt*_ (%)
Spot 1 (LOMs)	82.98	91.01	19.14
Spot 2 (HOMs)	74.22	96.45	21.19

### B. Comparison of computed and target fields and intensities

The efficiency, *η*_*dig*_ = *η*(*E*_*dig*_) for digitally computed output fields w.r.t the target fields, and the fidelity, *C*_*dig*_ = *C*(*I*_*dig*_*, I*_*target*_) and *RMSE*_*dig*_ = *RMSE*(*I*_*dig*_*/I*_*target*_) calculations for the digitally computed output intensities, *E*_*dig*_ or *I*_*dig*_ w.r.t the target intensities are summarized in Table II.

**TABLE II:** Efficiency calculations for the digitally computed fields w.r.t to the target fields and fidelity and RMSE calculations for digitally computed intensities w.r.t the target intensities.

Focal spot	η_*dig*_ (%)	*C*_*dig*_ (%)	*RMSE*_*dig*_ (%)
Spot 1 (AMs)	66.98	90.02	35.87
Spot 1 (LOMs)	75.93	84.69	44.58
Spot 2 (AMs)	64.54	95.96	38.29
Spot 2 (HOMs)	64.55	96.02	38.27

We observe from the table above that the efficiency marginally improves by selecting LOMs for the near axis focal spot while it barely changes for the focal spot at the boundary. This is an expected observation as we have created focal spots with mode control in their optimal regions. The efficiency curves in Figure 4 (a, b) also demonstrate this effect. On the other hand, the fidelity decreases, and the root mean square error increases as a result of mode selection. These observations are also explained by the increase in the background speckle intensity due to the unselected modes.

### C. Comparison of experimental and computed field and intensities

We can compare the digitally computed and experimental intensities using their cross fidelity, *C*_*dig−expt*_ = *C*(*I*_*dig*_*, I*_*out*_) and *RMSE*_*dig−expt*_ = *RMSE*(*I*_*dig*_*/I*_*out*_). These calculations are summarized in Table III.We observe very high, albeit not perfect correlation between the digitally computed and the experimentally obtained mode-controlled field.
